# Supplementary figures and images for: Ecological factors that drive microbial communities in culturally diverse fermented foods
Source: BMC Microbiol. 2025 Oct 15;25:655. doi: 10.1186/s12866-025-04413-6 (PMC12523157; doi:10.1186/s12866-025-04413-6)

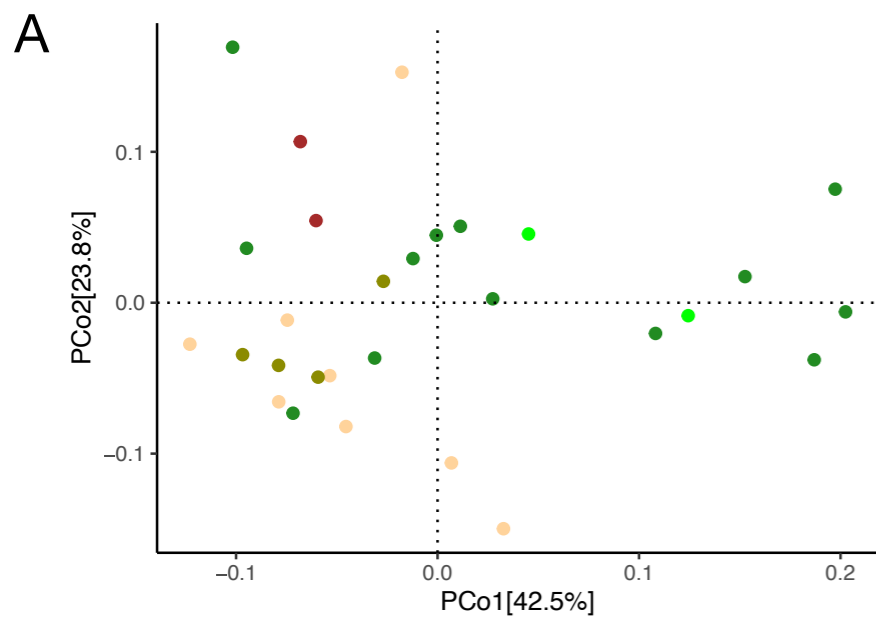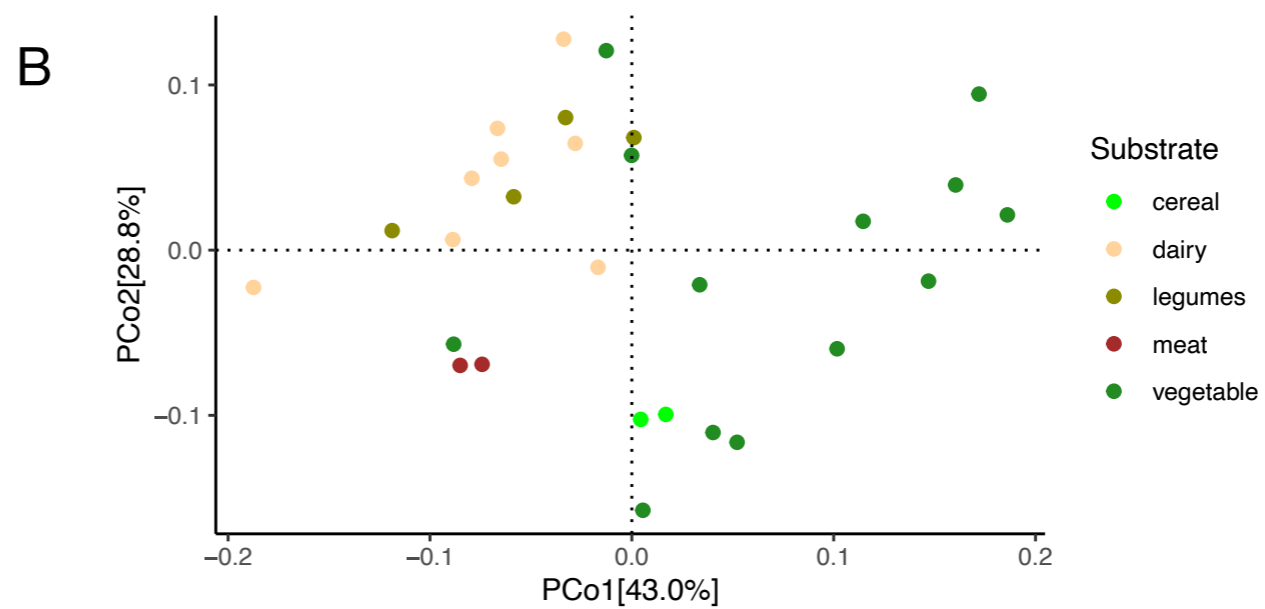

All taxa Canonical fermenters only

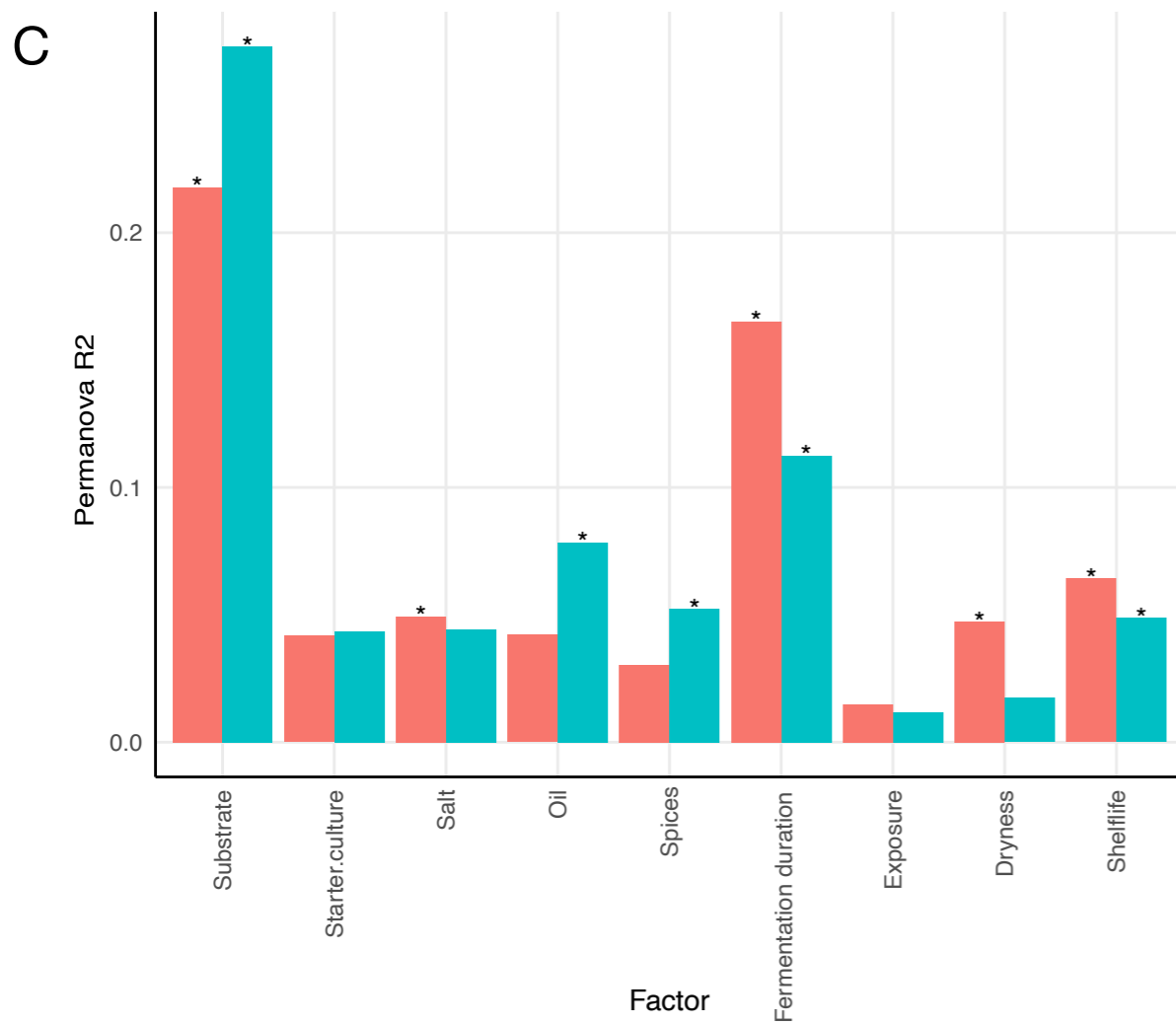

Supplement: Supplementary file 5 — Supplementary Material 5. [file 12866_2025_4413_MOESM5_ESM.pdf]

A

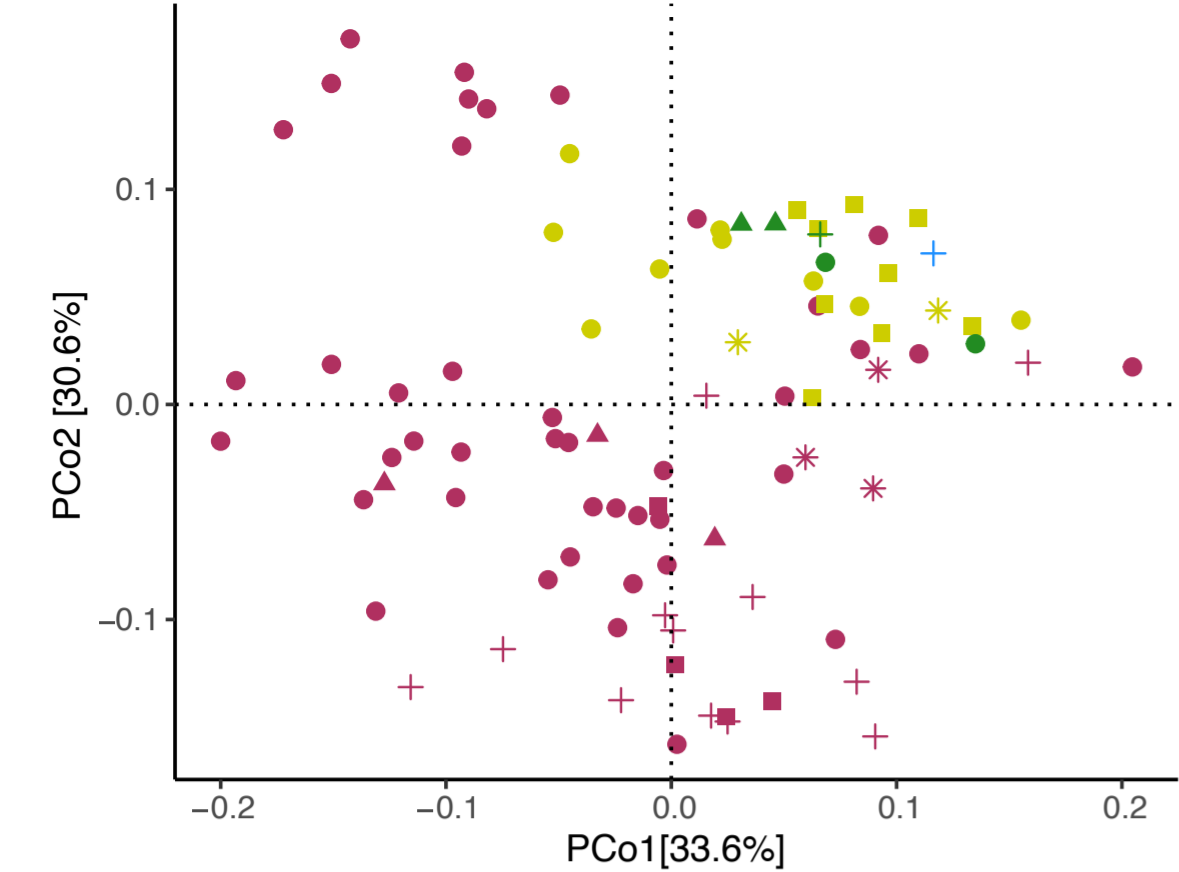

B

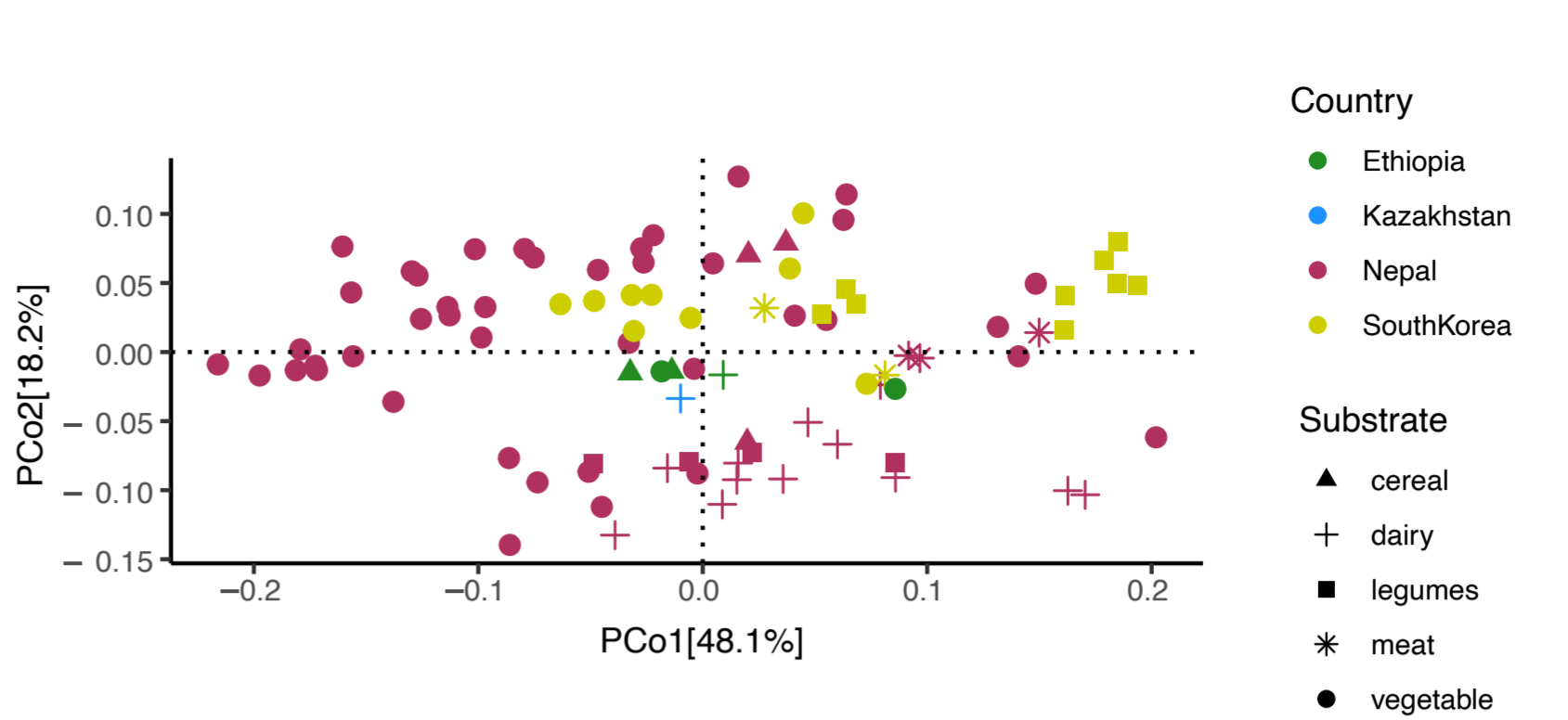

C

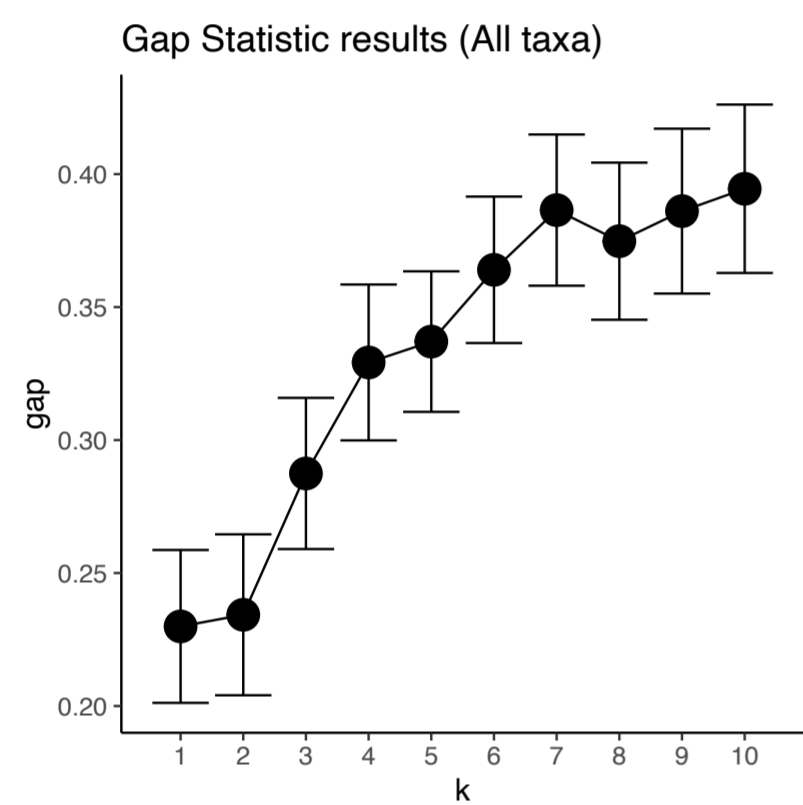

D

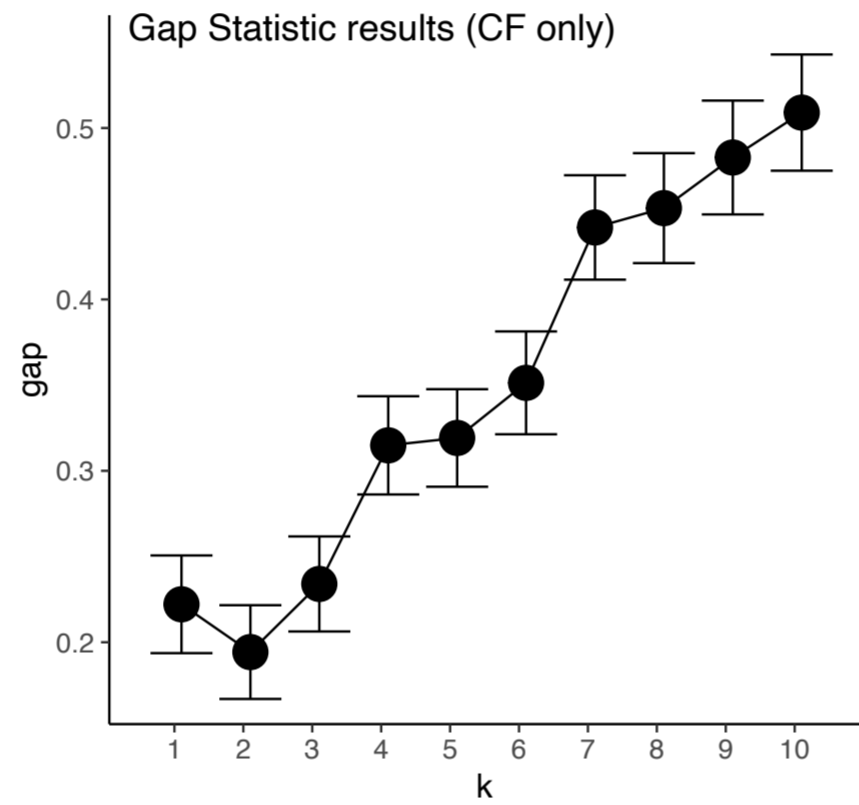

E

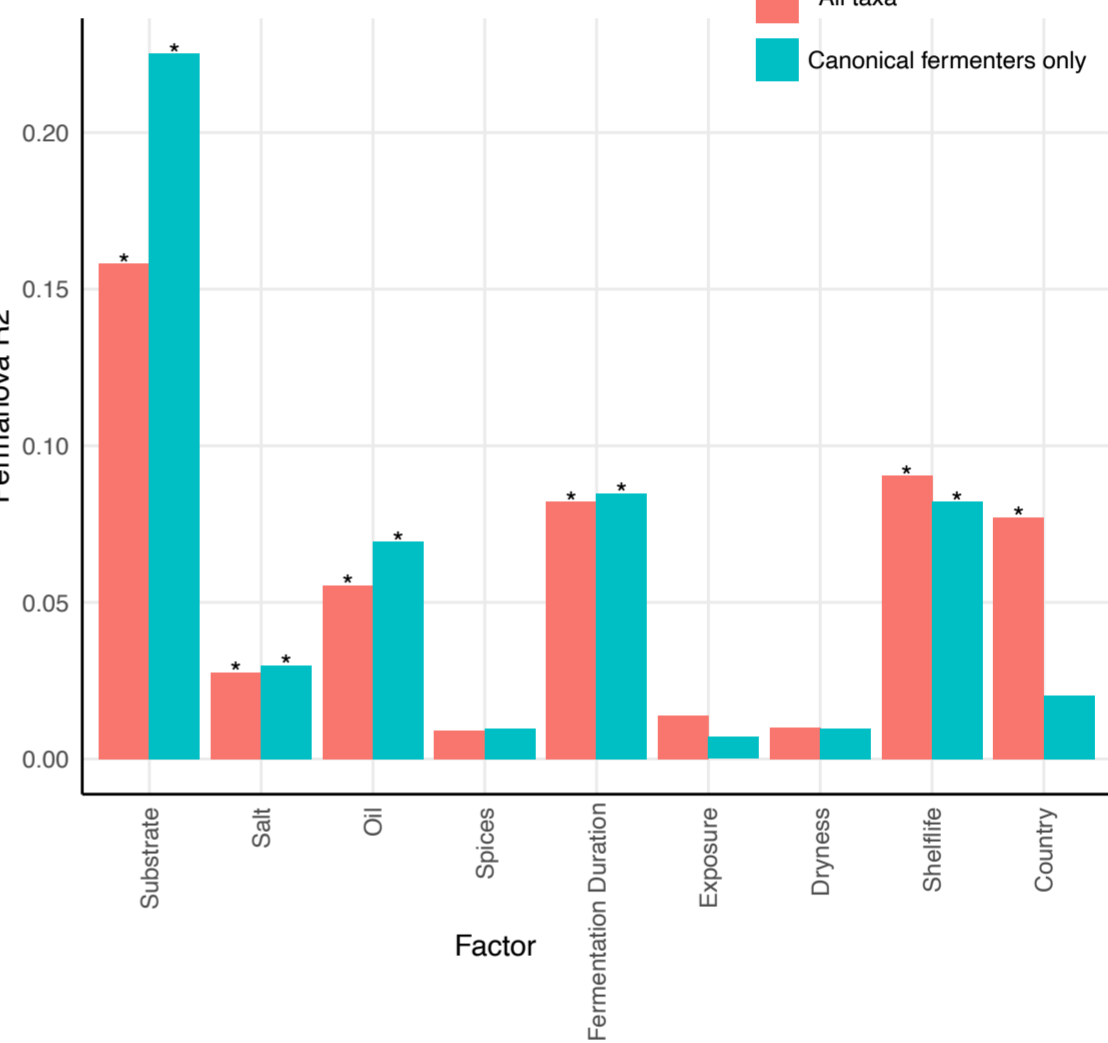

F

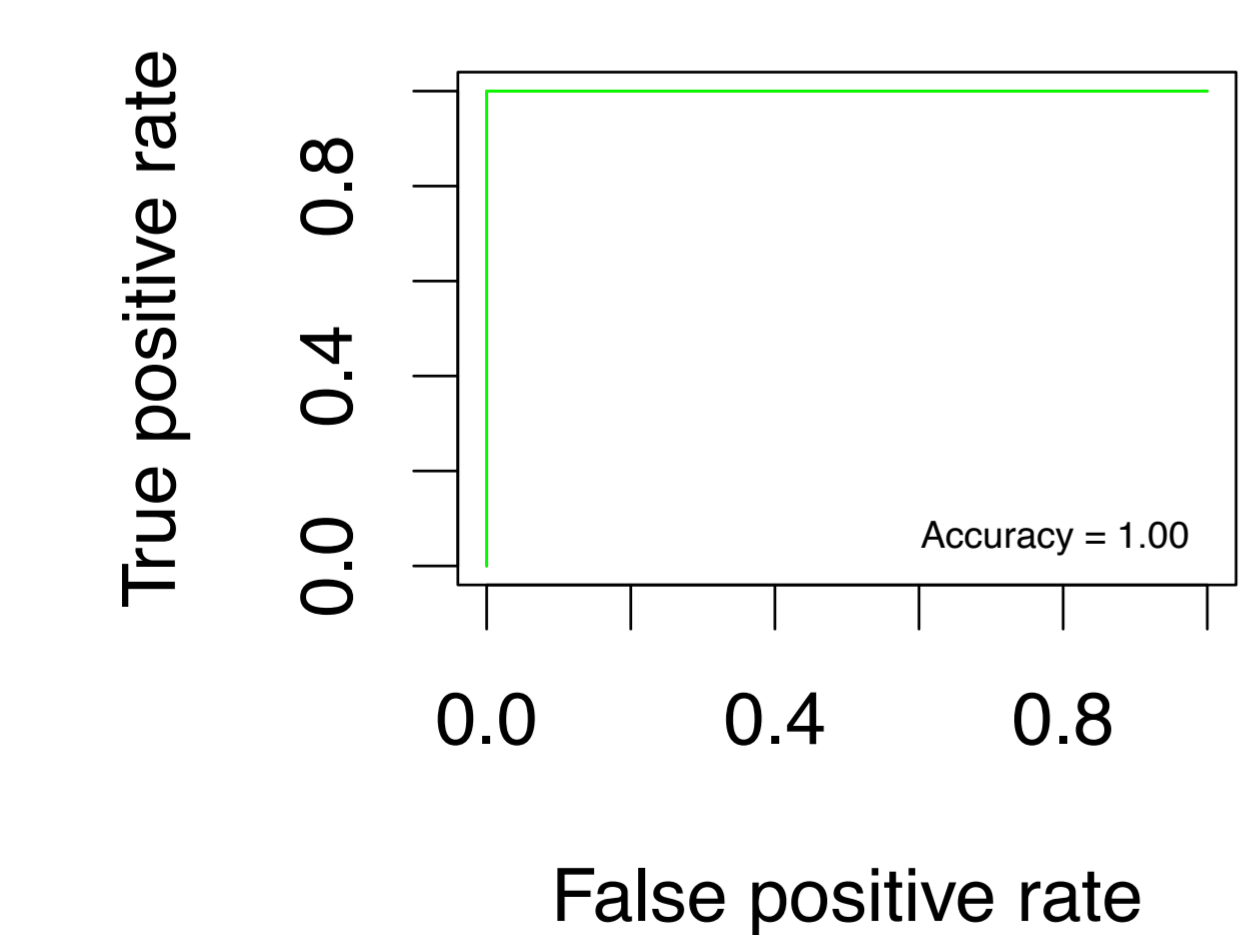

G

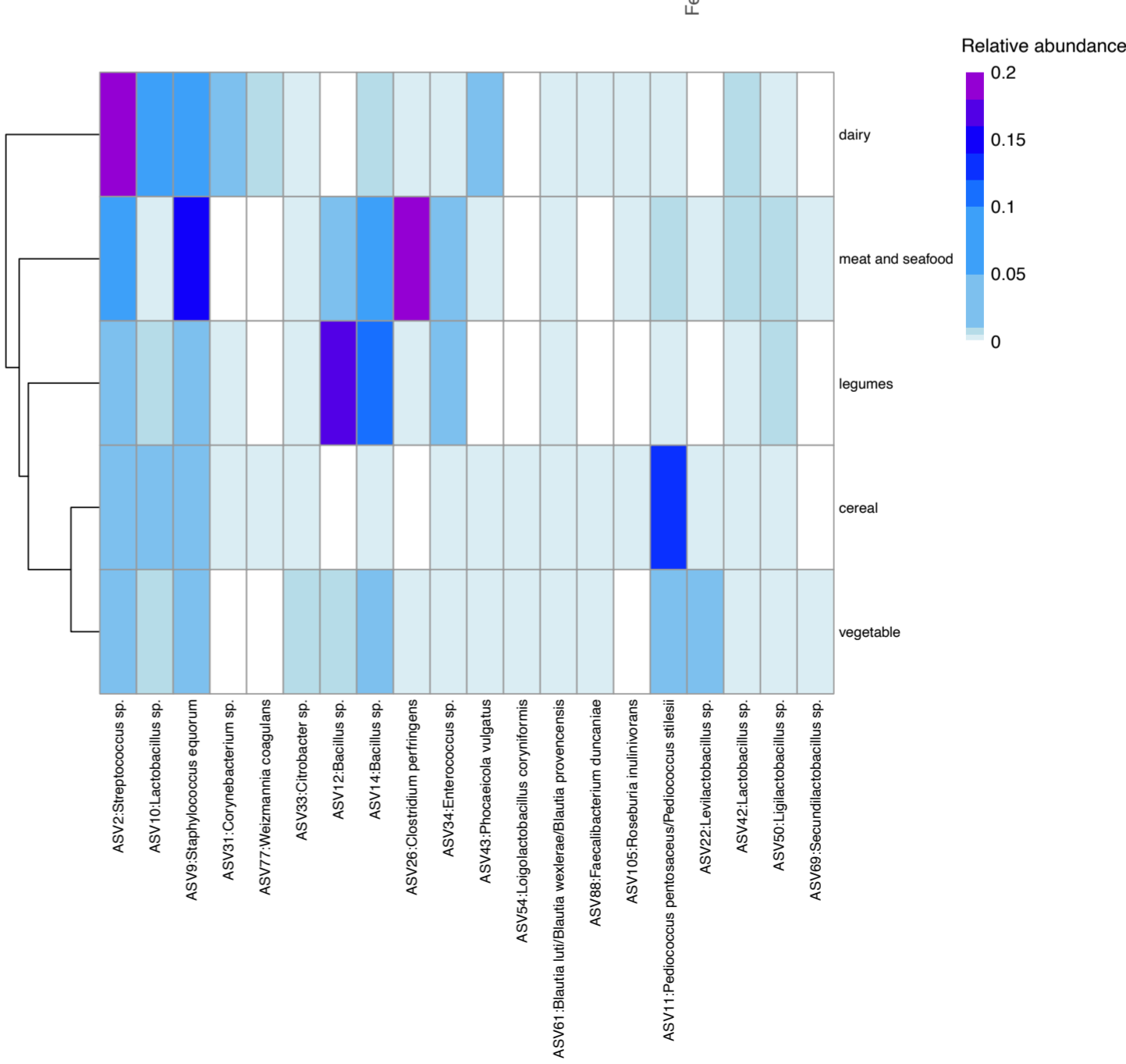

Supplement: Supplementary file 6 — Supplementary Material 6. [file 12866_2025_4413_MOESM6_ESM.pdf]

A

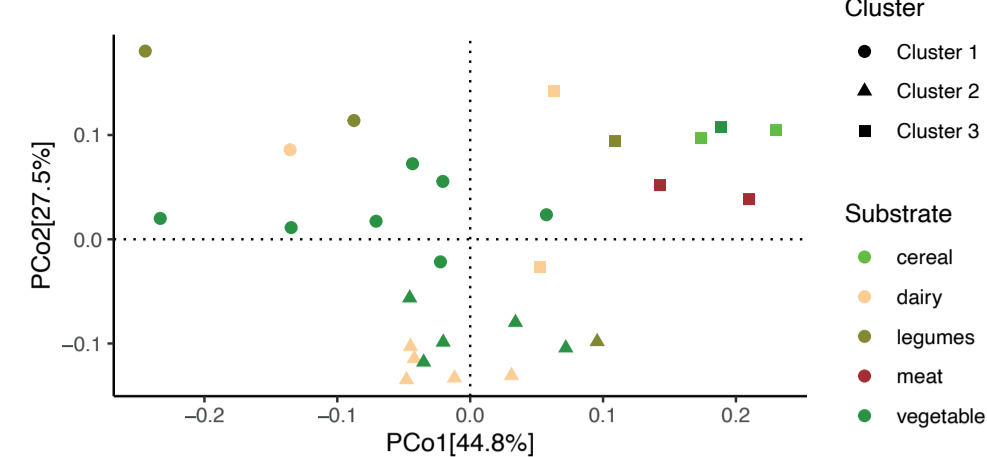

B

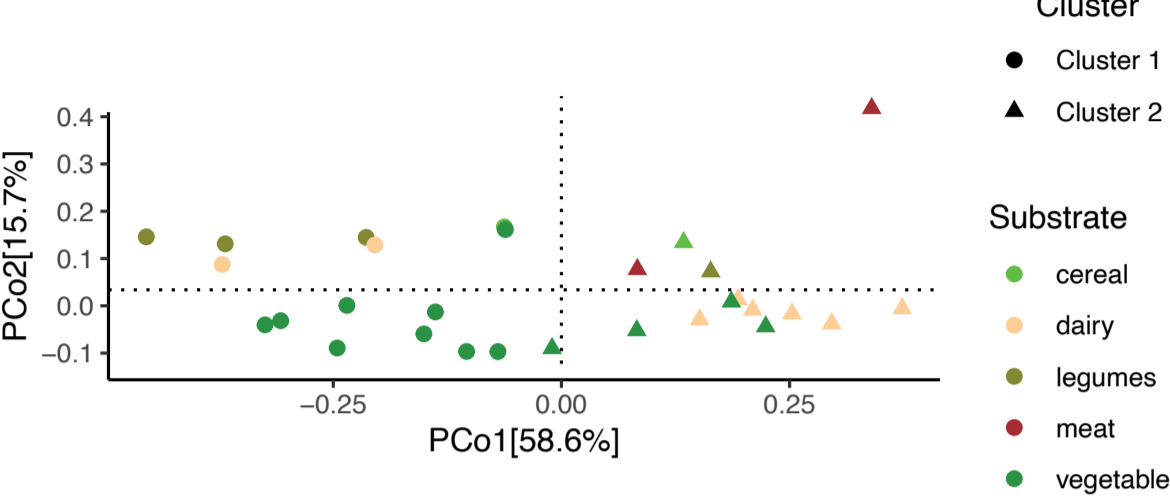

C

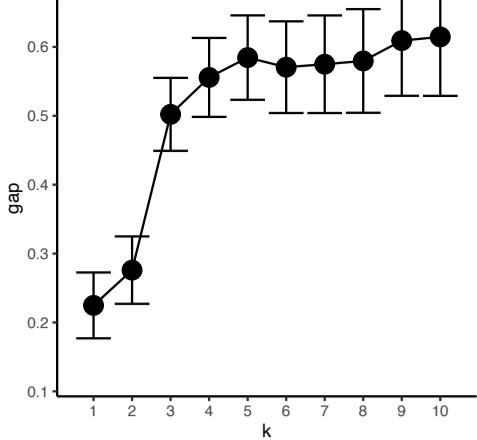

D

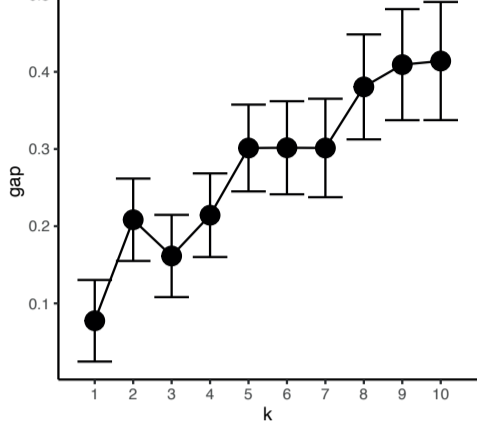

E

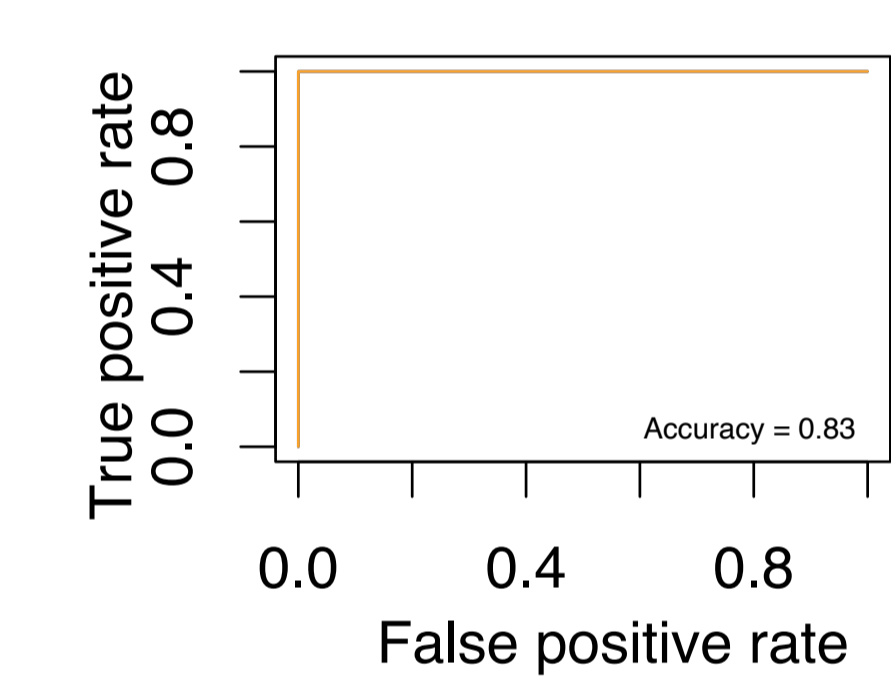

F

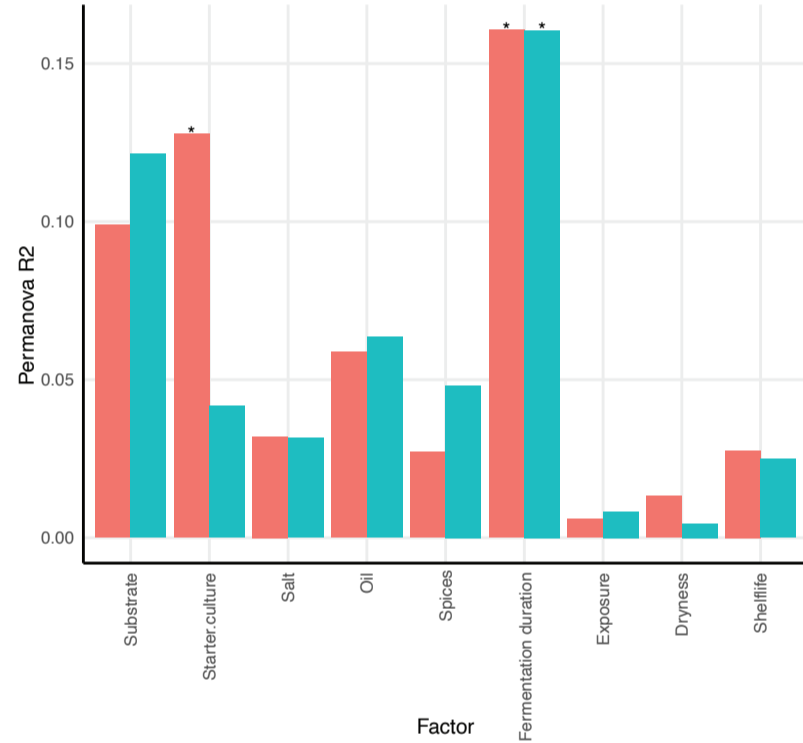

G

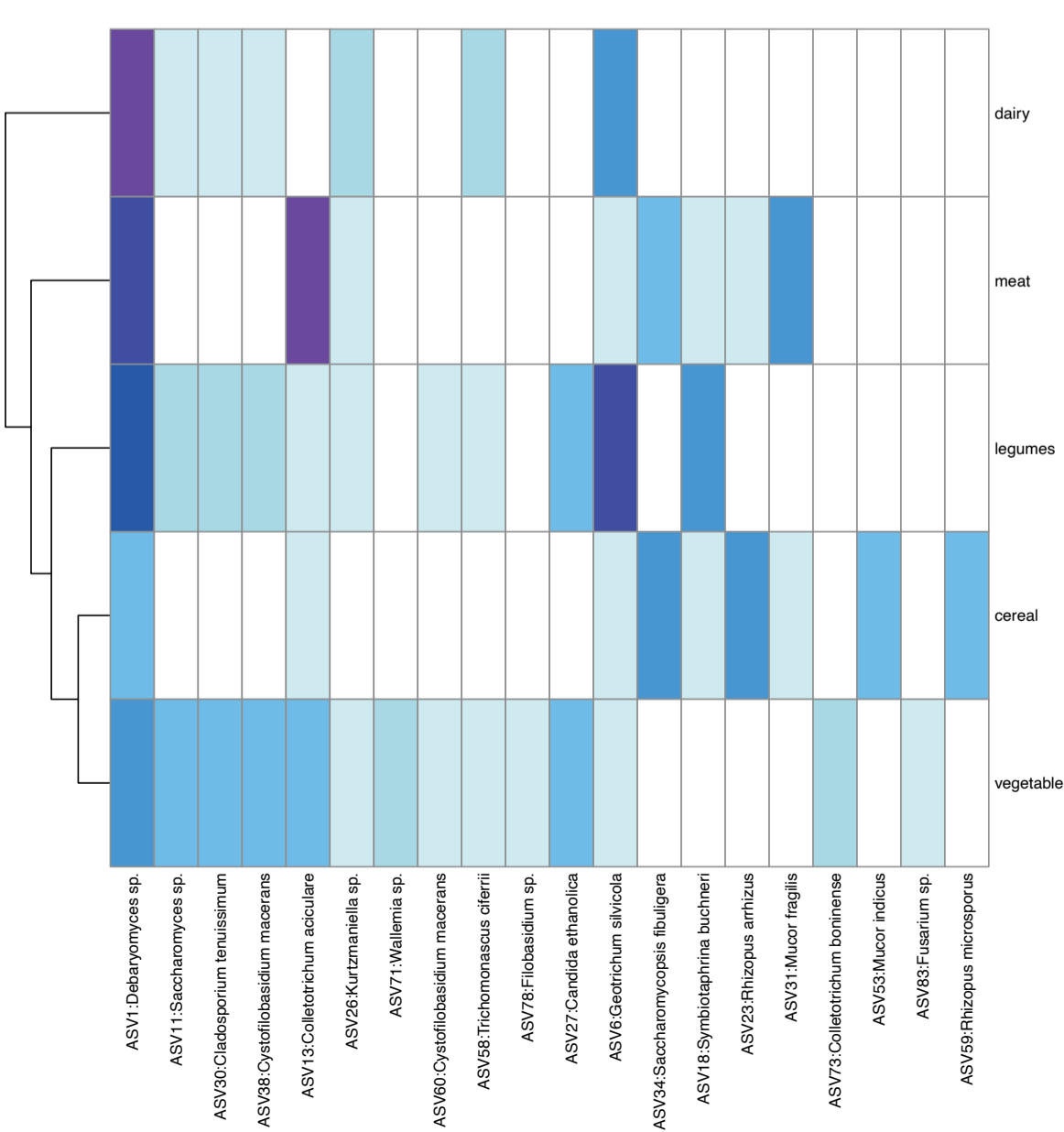

Supplement: Supplementary file 7 — Supplementary Material 7. [file 12866_2025_4413_MOESM7_ESM.pdf]

A

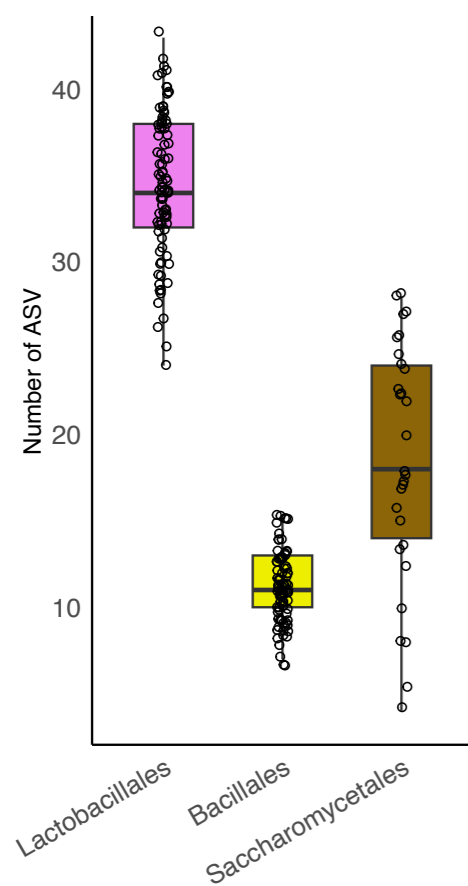

B

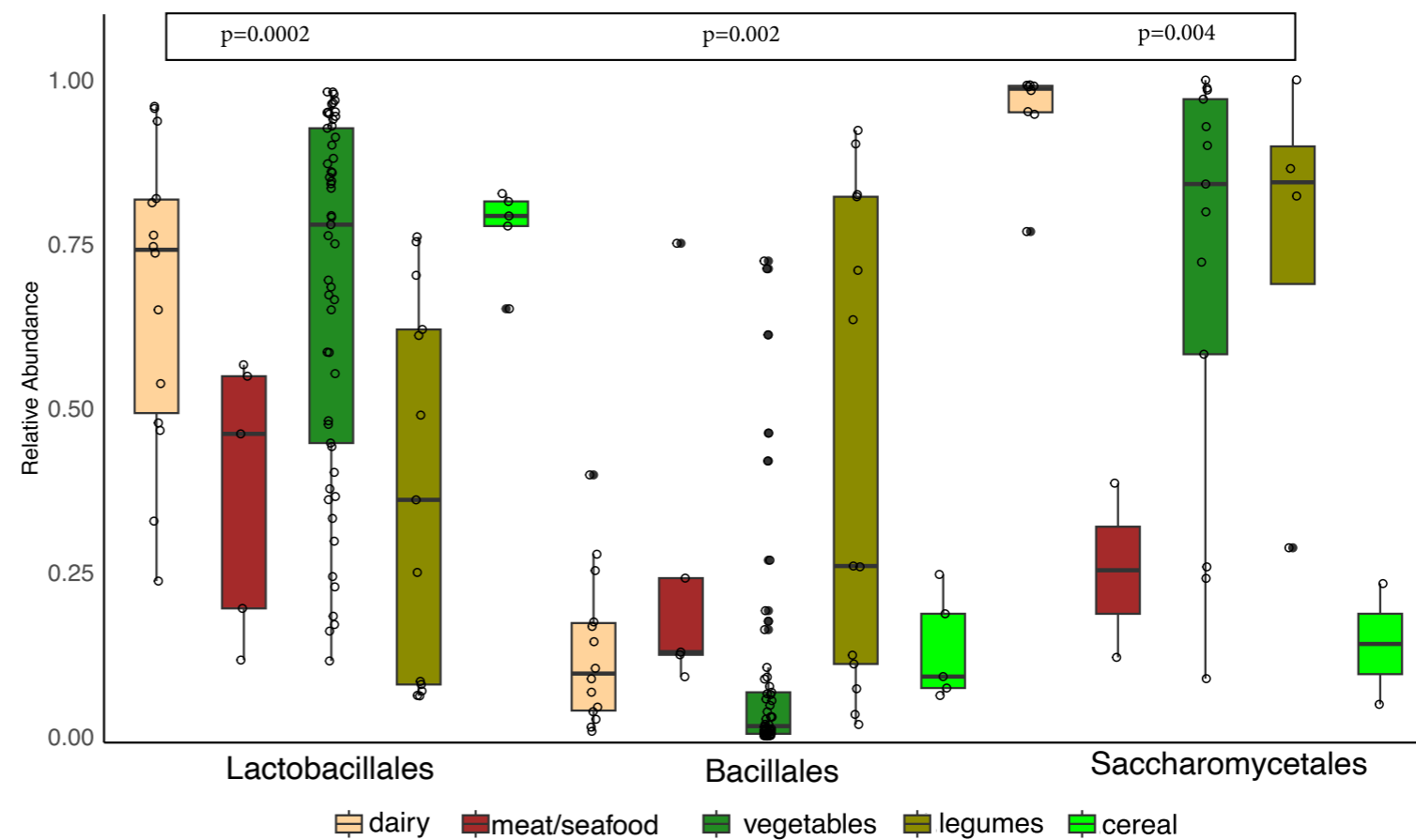

C

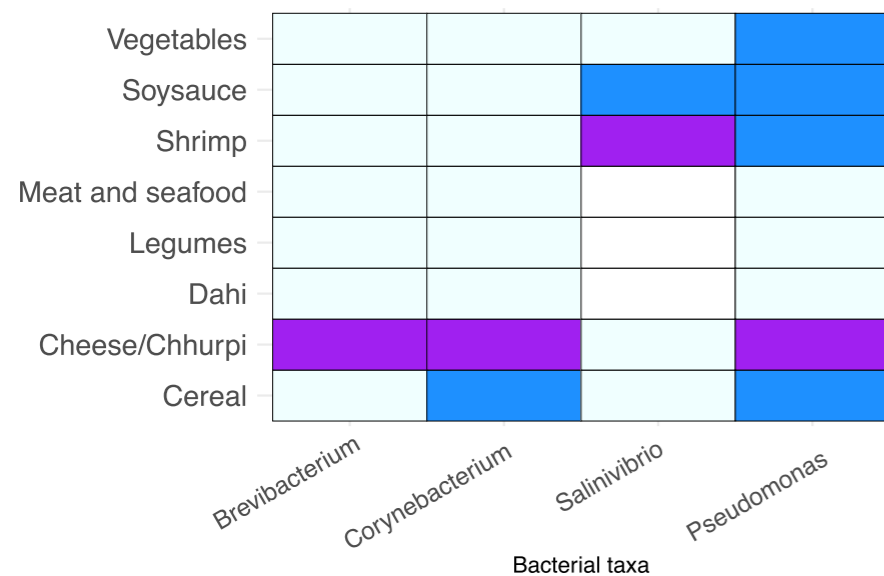

D

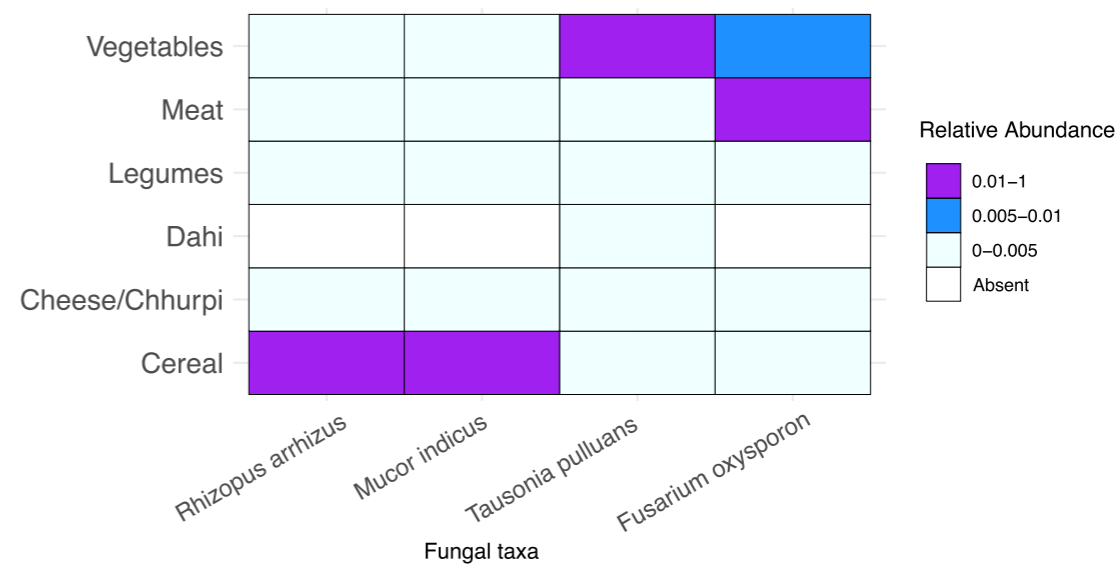

Supplement: Supplementary file 8 — Supplementary Material 8. [file 12866_2025_4413_MOESM8_ESM.pdf]

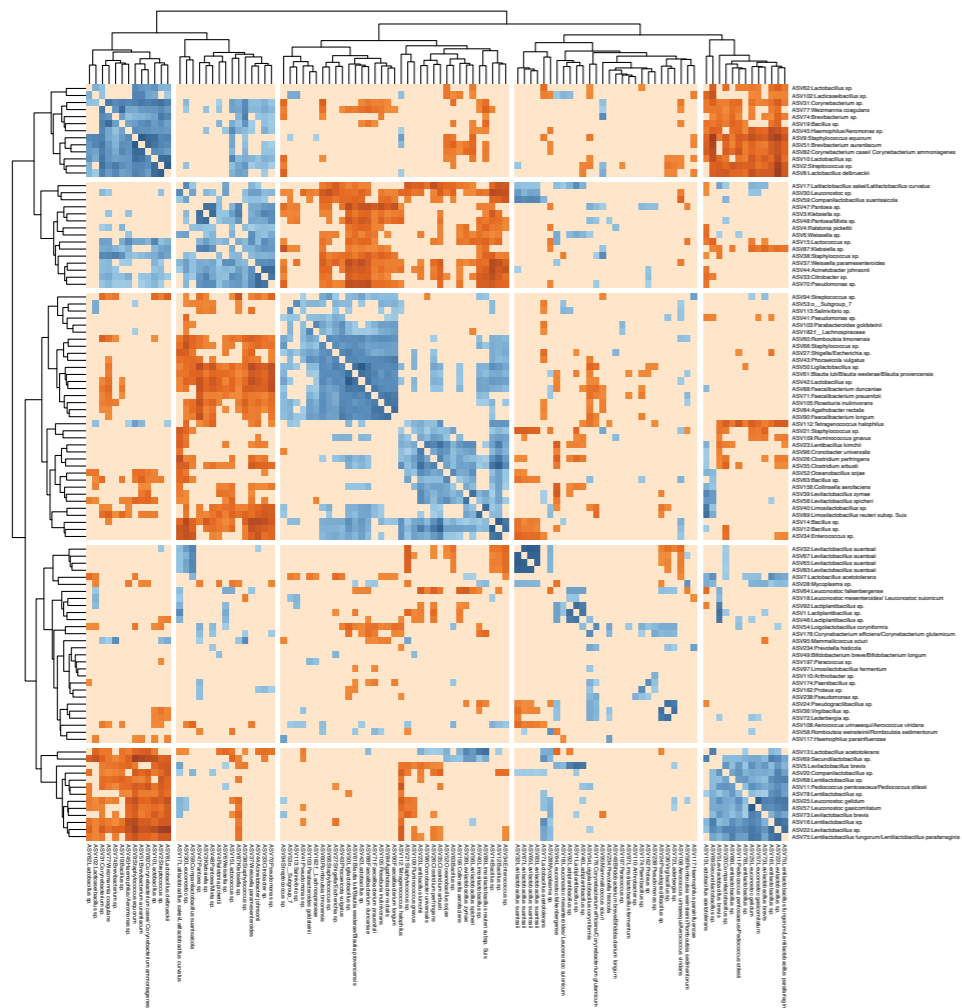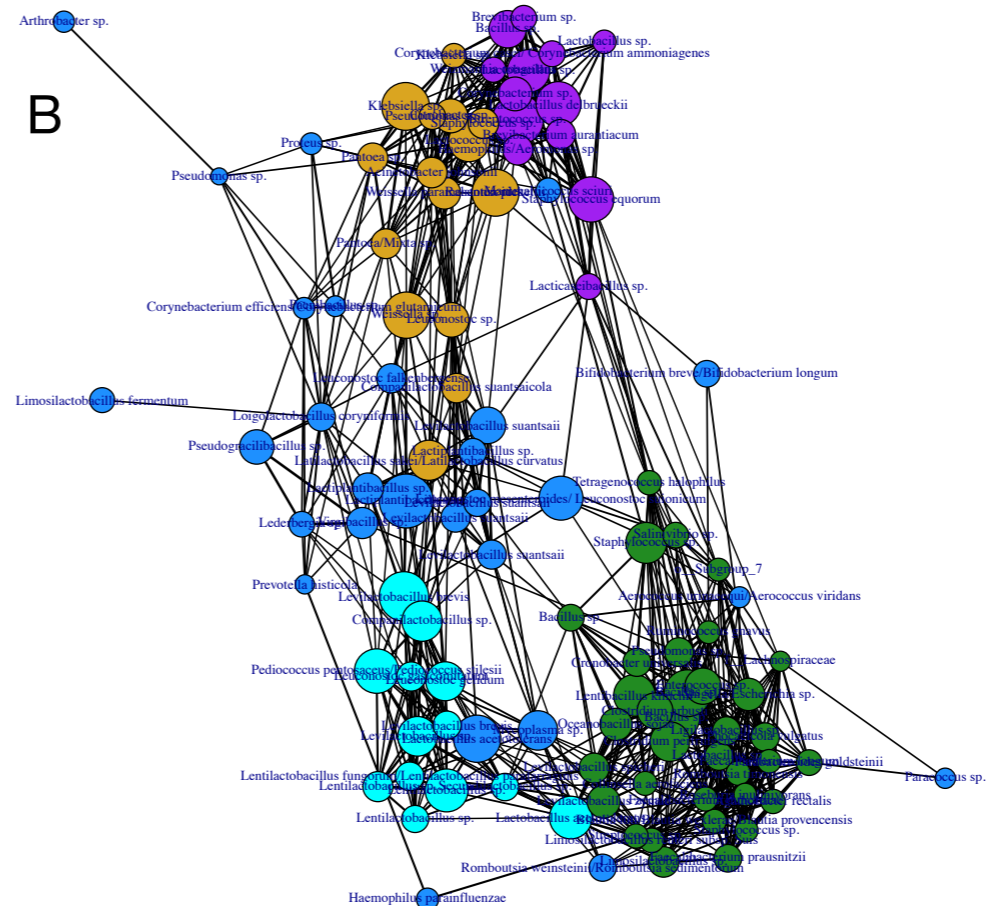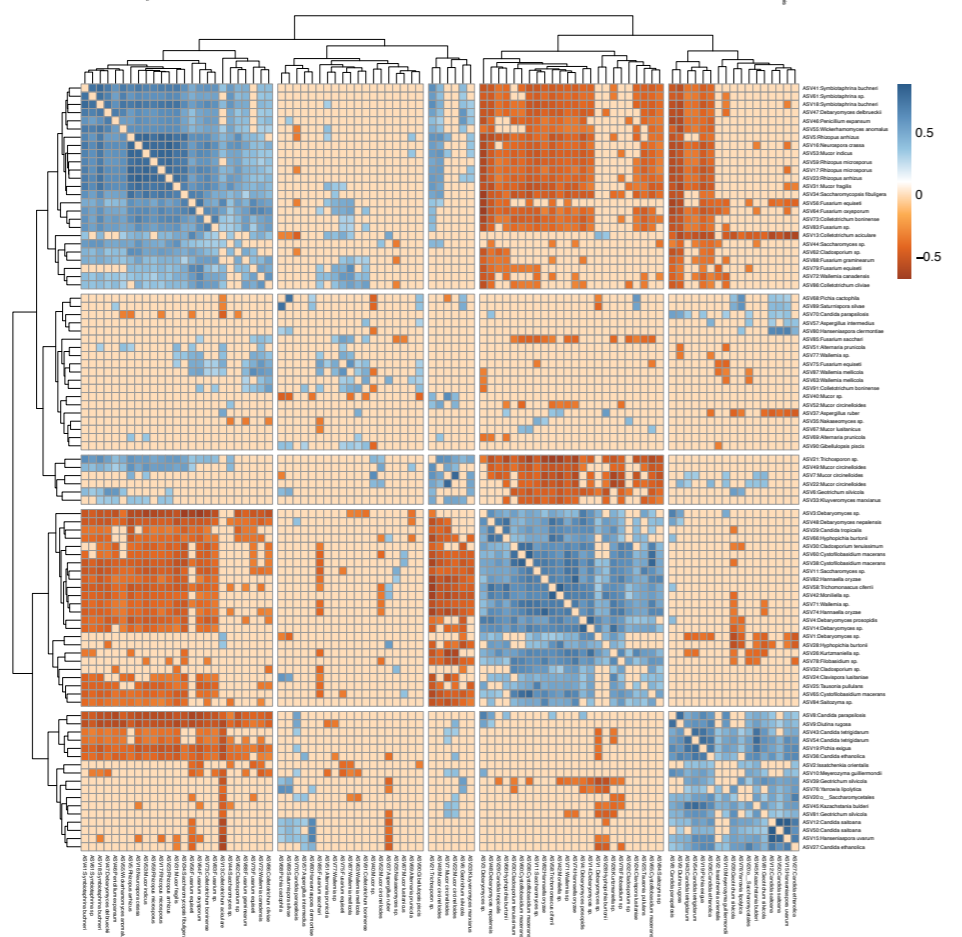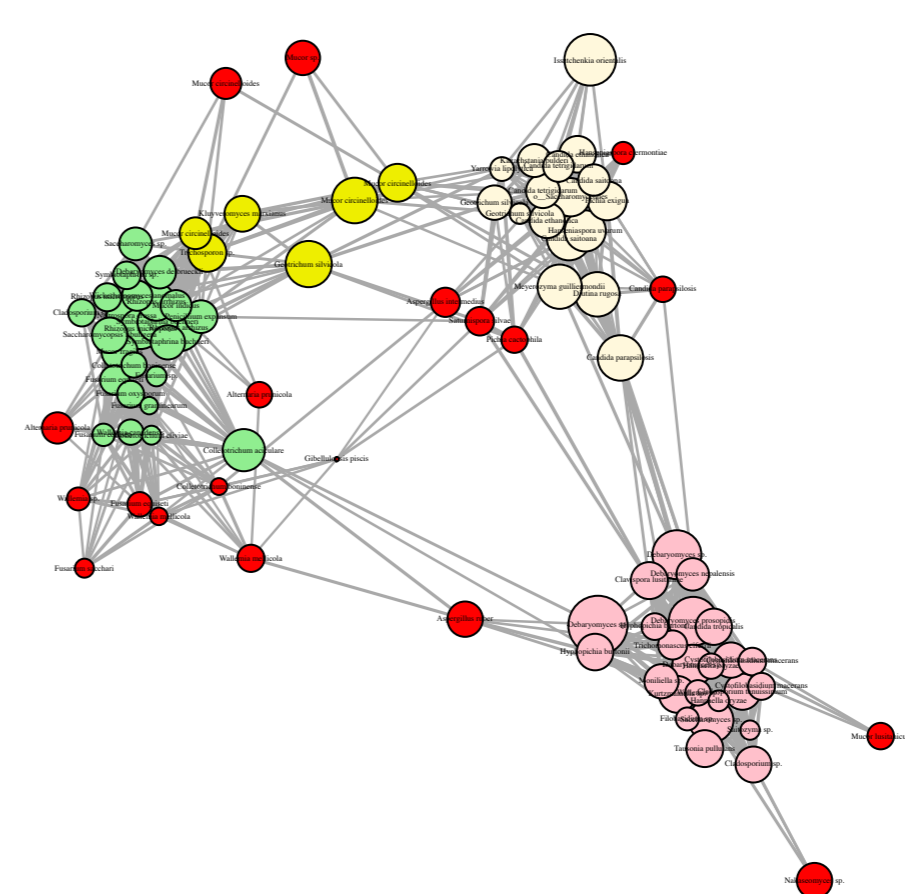

Supplement: Supplementary file 9 — Supplementary Material 9. [file 12866_2025_4413_MOESM9_ESM.pdf]

A

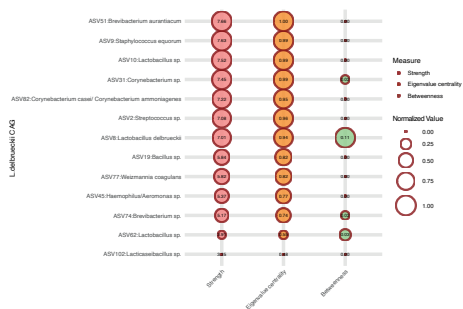

B

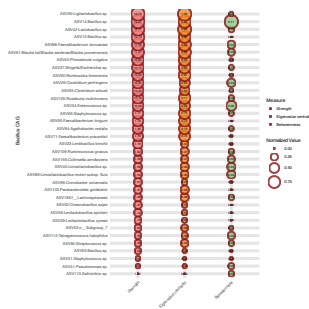

C

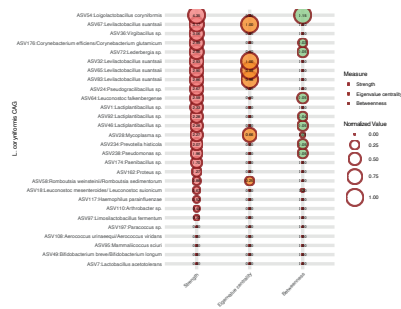

D

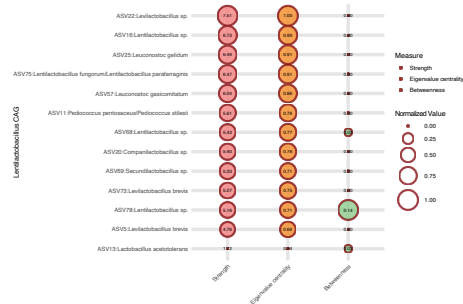

E

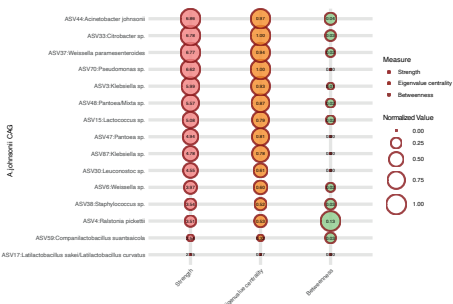

F

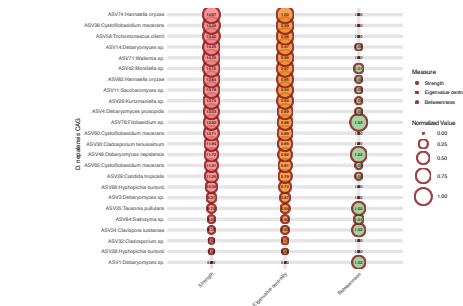

G

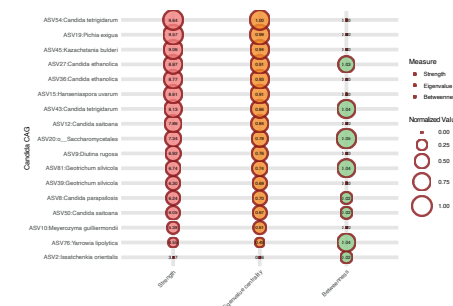

H

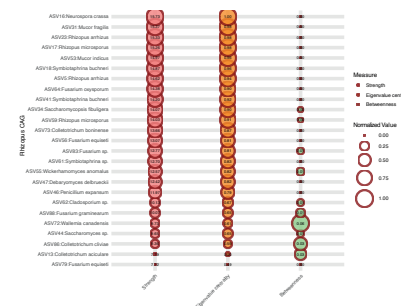

I

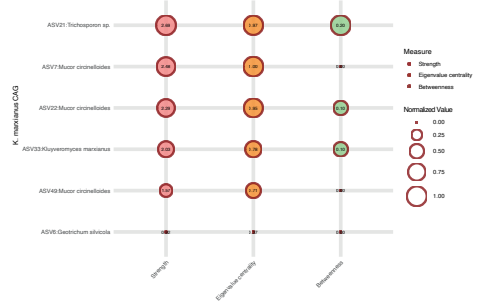

J

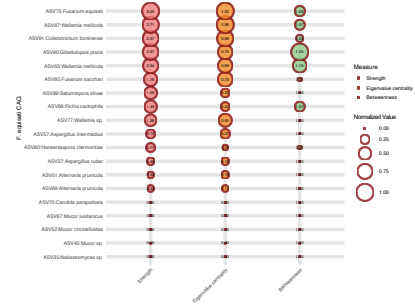

Supplement: Supplementary file 10 — Supplementary Material 10. [file 12866_2025_4413_MOESM10_ESM.pdf]

A

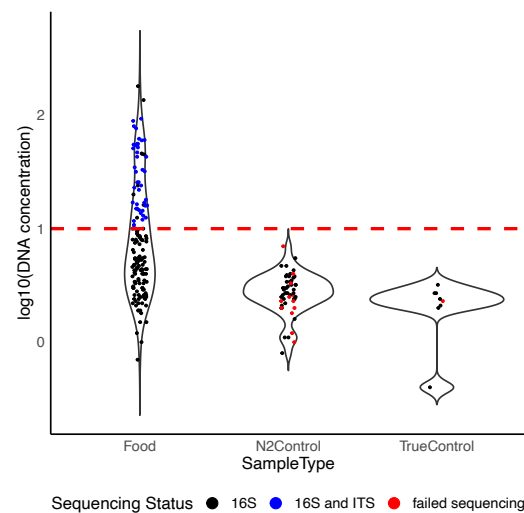

B

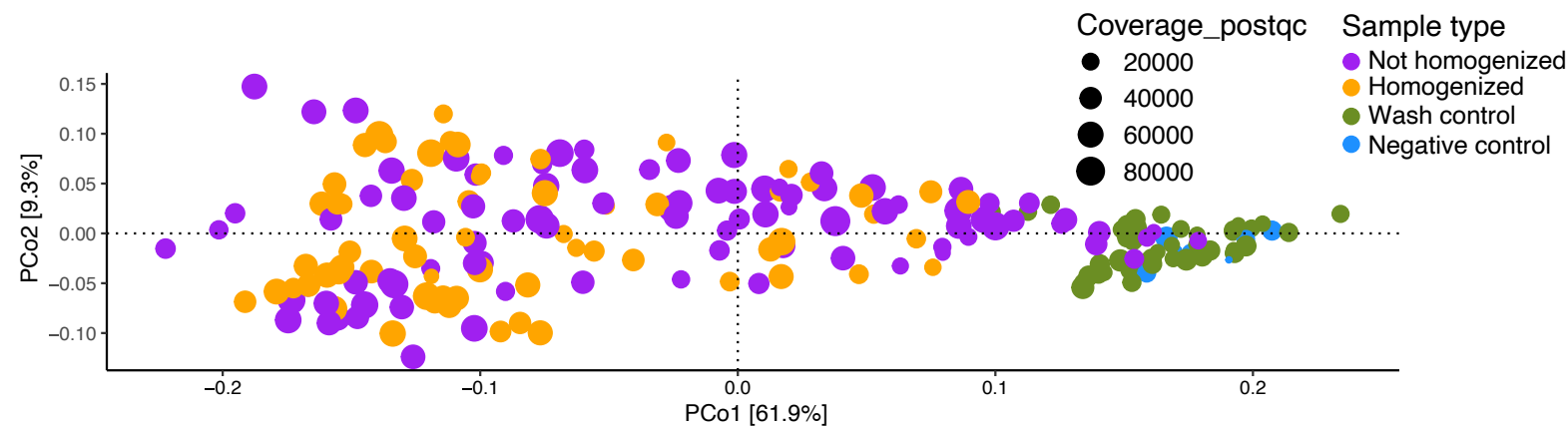

C

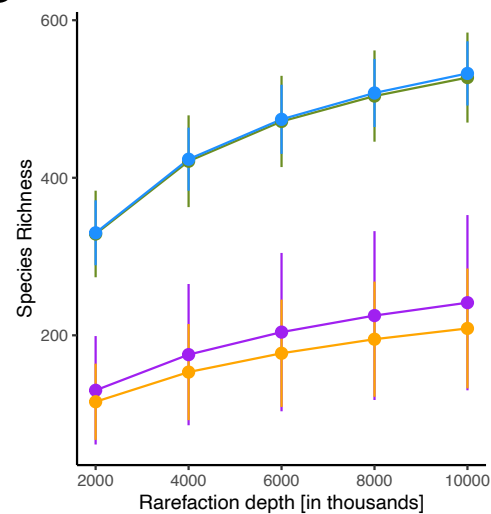

D

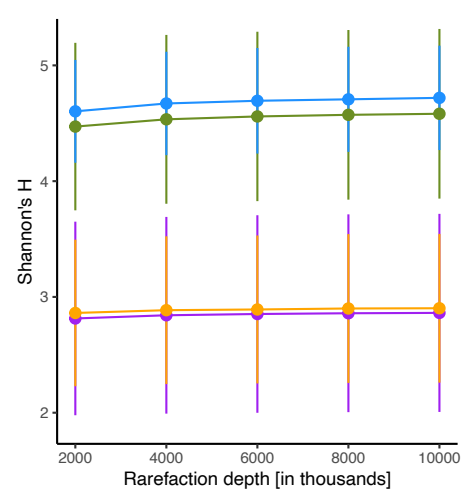

E

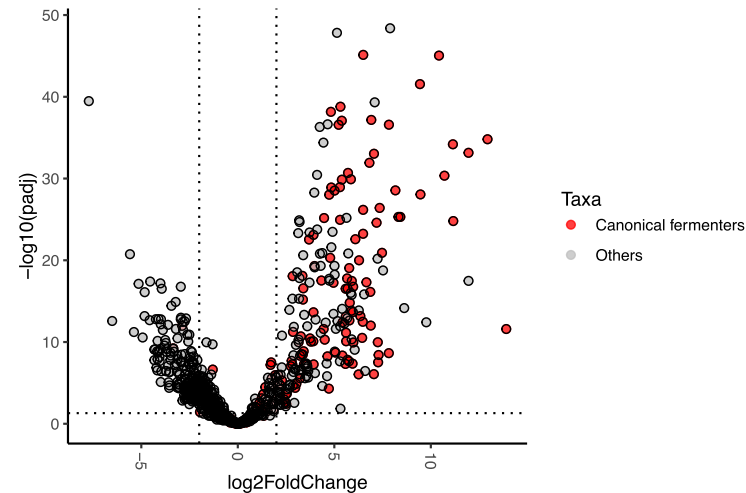

F

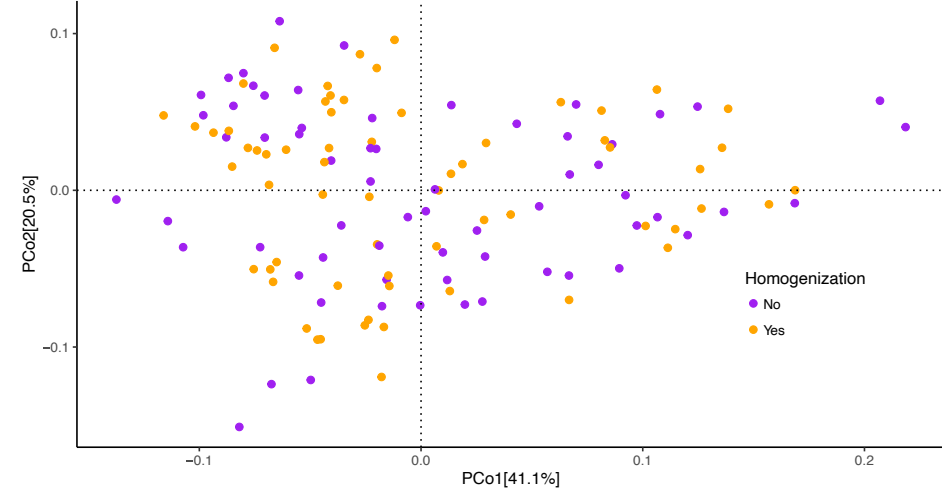

G

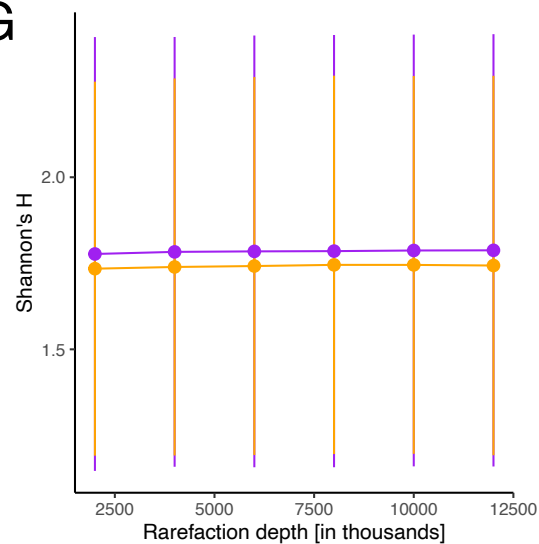

H

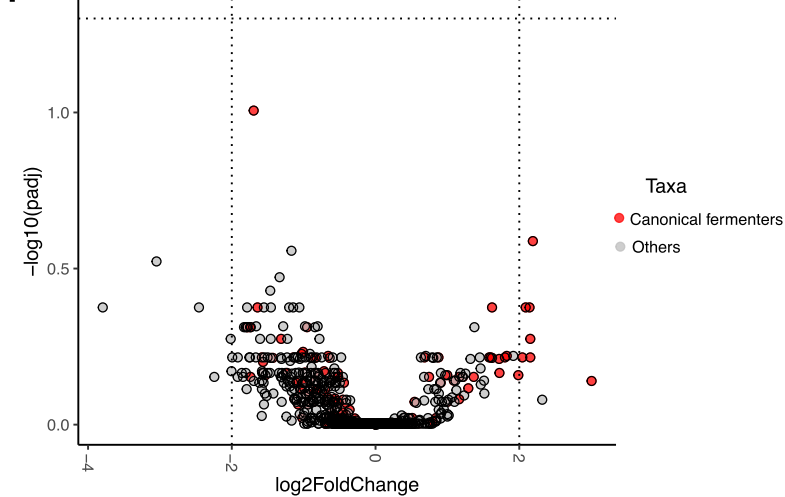

I

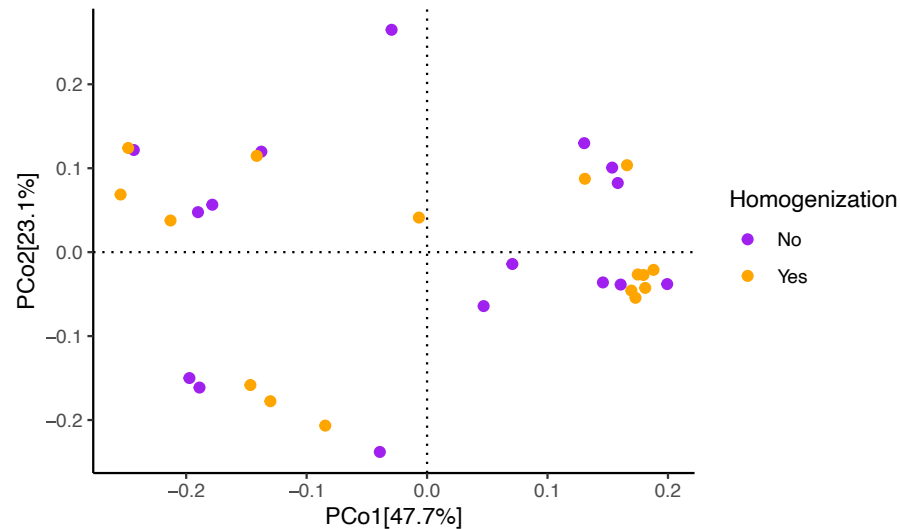

J

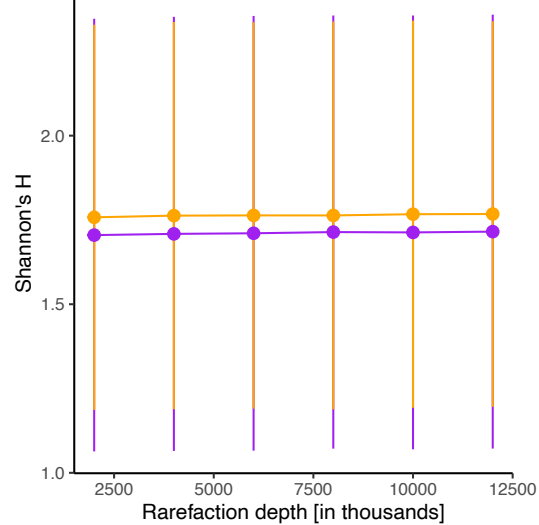

K

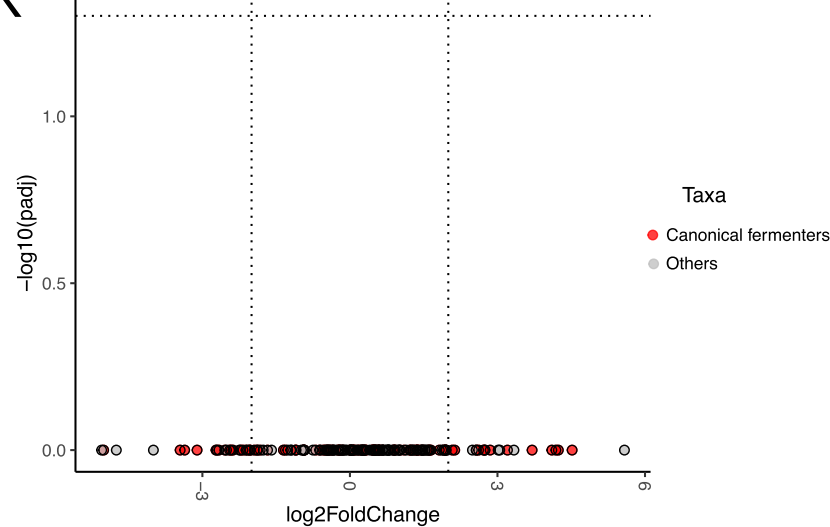

Supplement: Supplementary file 11 — Supplementary Material 11. [file 12866_2025_4413_MOESM11_ESM.pdf]

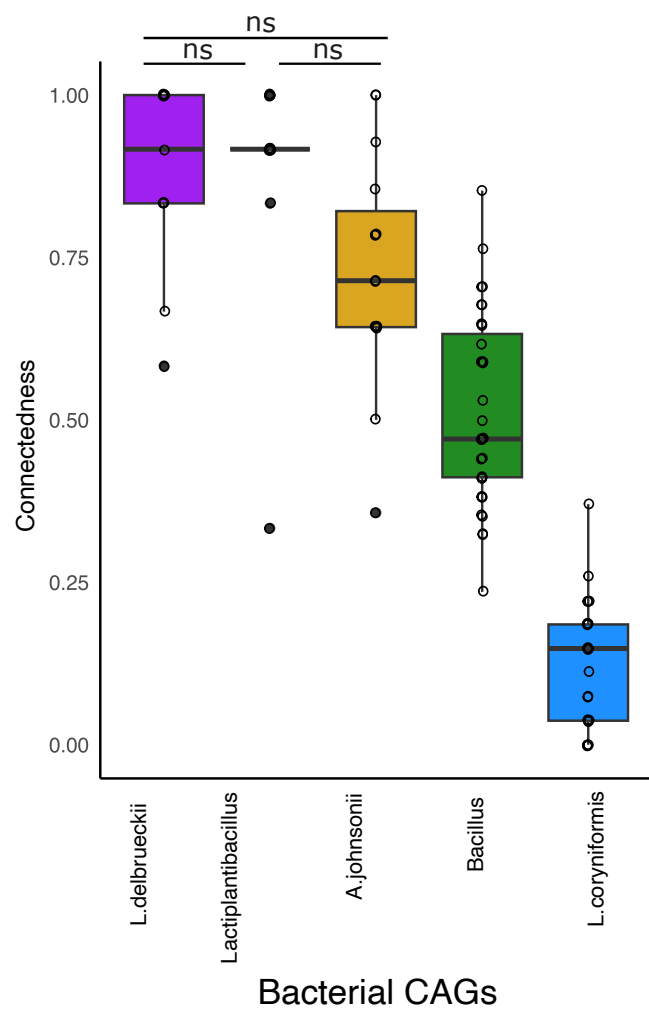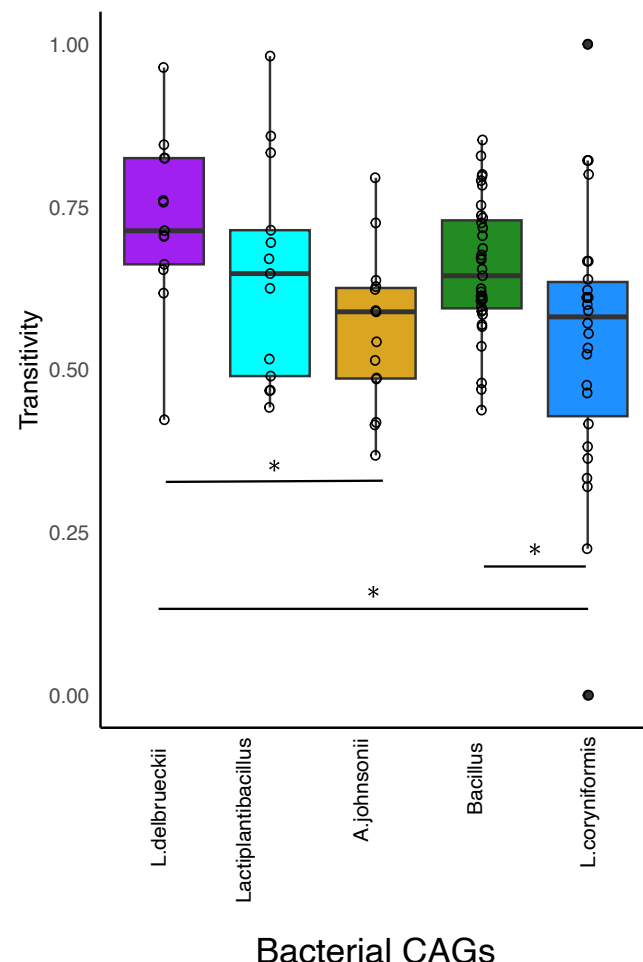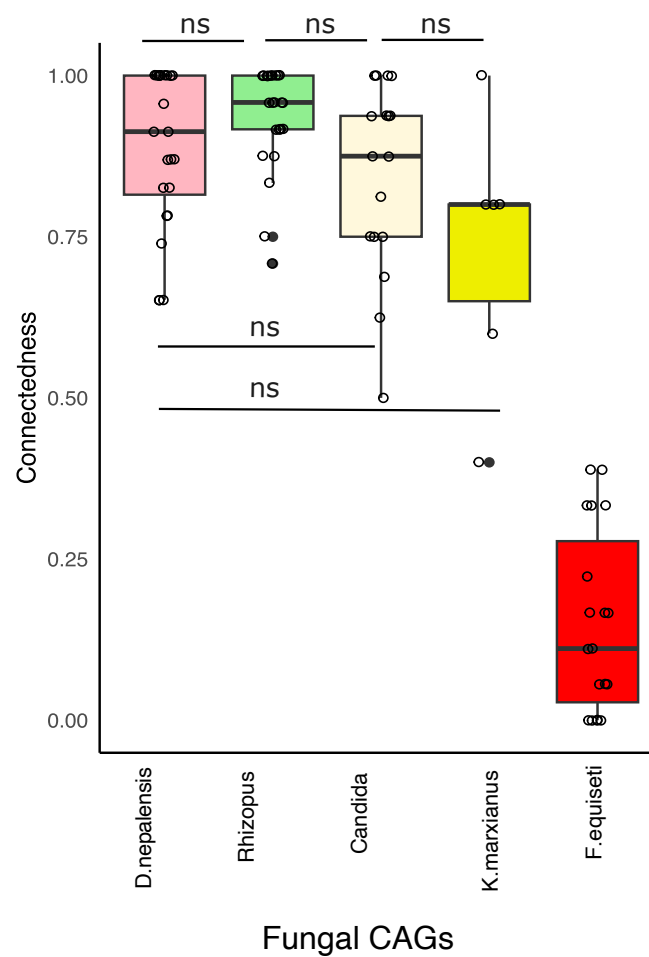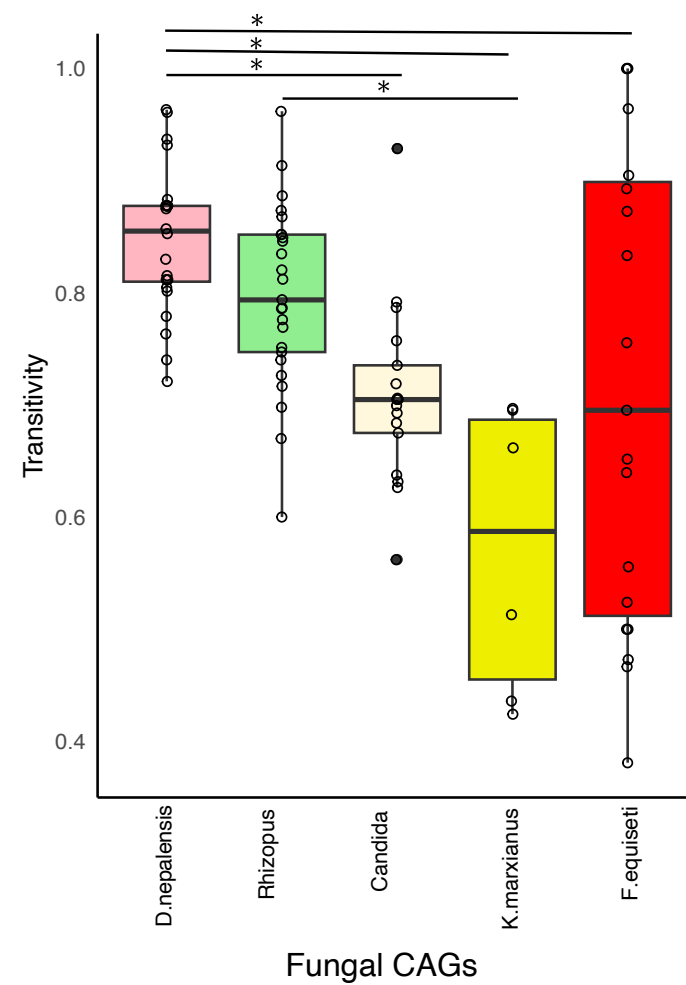

Supplement: Supplementary file 12 — Supplementary Material 12. [file 12866_2025_4413_MOESM12_ESM.pdf]
